# Pre-exposure prophylaxis uptake for high-risk men who have sex with men in China: a multi-city cross-sectional survey

**DOI:** 10.1186/s12981-023-00528-w

**Published:** 2023-06-02

**Authors:** Guang Zhang, Xue Yang, Wenting Kang, Tongtong Liu, Lili Cheng, Meixia Qu, Xinlun Wang, Houlin Tang

**Affiliations:** 1 National Center for AIDS/STD Control and Prevention, Chinese Center for Disease Control and Prevention, Beijing, 102206 China; 2 Beijing Huilongguan Hospital, Beijing, 100096 China; 3 Chinese Association of STD and AIDS Prevention and Control, Beijing, 100050 China; 4 Chinese Health Education Center, Beijing, 100011 China

**Keywords:** HIV, Pre-exposure prophylaxis, Men who have sex with men

## Abstract

**Background:**

Pre-exposure prophylaxis (PrEP) is a proven biomedical strategy to prevent HIV transmission among men who have sex with men (MSM). Despite oral PrEP is safe and effective in MSM, the use of PrEP has been discouraging, especially in high-risk MSM. And there are no relevant studies showing the use of PrEP in high-risk MSM. The purpose of this study was to get the rate of PrEP use and the factors influencing PrEP use among high-risk MSM.

**Methods:**

A cross-sectional study was conducted through an electronic questionnaire on the “i guardian Platform”, and “snowballing” method was used to recruit MSM in six cities in China, including Beijing, Shenzhen, Chengdu, Changsha, Jinan and Nanjing in China, from January to April 2021. Univariate and multivariate logistic regression analysis were used to analyze the factors associated with the use of PrEP among high-risk MSM who had heard about PrEP.

**Results:**

Among the 1865 high-risk MSM who had heard of PrEP, the rates of those who were willing to use PrEP, had knowledge awareness of PrEP, and had used PrEP were 96.7%, 24.7%, and 22.4%, respectively. Multivariate logistic regression analysis of PrEP use in high-risk MSM showed that more PrEP was used by those who were 26 years or older (*OR* = 1.86, 95%*CI* 1.17 ~ 2.99), had master degree or above (*OR* = 2.37, 95% *CI* 1.21 ~ 4.72), had unstable work (*OR* = 1.86, 95% *CI* 1.16 ~ 2.96), had tested five or more HIV times in the past year (*OR* = 3.09, 95% *CI* 1.65 ~ 6.04), had consulted PrEP (*OR* = 22.05, 95% *CI* 14.87 ~ 33.91) and had PrEP knowledge awareness (*OR* = 1.90, 95% *CI* 1.41 ~ 2.55) (*P* < 0.05).

**Conclusions:**

The rate of PrEP use in high-risk MSM was relatively low. PrEP was used more by high-risk MSM with unstable jobs, higher education, frequent HIV testing, and PrEP counseling. Public education on PrEP for MSM should continue to be enhanced to help them use PrEP in a timely and accurate manner.

## Introduction

By the end of 2020, there were 1.053 million people living with human immunodeficiency virus (HIV) in China, and the cumulative number of reported deaths was 351,000 [1]. Data extracted from 355 studies in China estimated the overall prevalence of HIV among men who have sex with men (MSM) from 2001 to 2018 was 5.7% [95% confidence interval (CI): 5.4% ~ 6.1%] [2]. MSM are a high-risk group for HIV infection and a growing public health challenge in China.

Pre-exposure prophylaxis (PrEP), a preventive measure to prevent HIV infection by taking antiviral drugs in people who are not yet infected but are at risk of infection, is one of the most important preventive measures for HIV [3]. World Health Organization (WHO) [4] recommends oral PrEP as an additional prevention strategy for key populations (including MSM), who are at high risk of HIV infection in 2012. In 2016, the Chinese Center for Disease Control and Prevention (CDC) released the Guidelines for HIV Prevention Interventions in High-Risk Populations, which for the first time clarified the applicable population, medication regimen and follow-up for PrEP in MSM [5]. Expert Consensus on PrEP for HIV prevention in China was published in 2020 [6]. In the same year, Truvada (oral PrEP medicine) was approved by the National Medical Products Administration of China in August in China [7]. Despite several policies emphasizing the importance of implementing PrEP among MSM in China, there are still no separate PrEP guidelines for MSM to directly refer to.

Despite oral PrEP is safe and effective in MSM, the use of PrEP has been discouraging, especially in MSM [8–10]. The U.S. National AIDS Strategy (2021) reported that 13.2% of MSM used PrEP in 2017, which was far from the goal that 50% of PrEP-eligible MSM should use PrEP [11]. In the Italian snowball survey, only 7.5% of MSM had used PrEP [12]. A cross-sectional survey in China based on online questionnaires showed that 4.3% of MSM had used PrEP [13]. Therefore, the rate of PrEP uptake in MSM is low and there are no relevant studies showing the use of PrEP in high-risk MSM. In addition, barriers to PrEP among gay, bisexual, and other MSM have received substantial research attention, but less is known about what factors may be affecting PrEP use among high-risk MSM [14].

Therefore, a cross-sectional study was designed to obtain information on the use of PrEP in high-risk MSM and the factors influencing PrEP use, in order to provide a theoretical basis for future PrEP guidelines in China.

## Methods

### Study design

A cross-sectional survey was conducted from January 2021 to April 2021 in six cities in China, including Beijing, Shenzhen, Chengdu, Changsha, Jinan and Nanjing. These six cities had high rates of new HIV infections in China (above 3/100 person-years) [15]. Given the strong influence of social organizations for MSM among gay in China, this study relied on them to distribute an electronic questionnaire to eligible MSM and then recruited more participants through the snowballing method [16].The inclusion criteria for participants were as follows. (1) completed informed consent; (2) male 18 years of age or older; (3) had sex with men; and (4) HIV negative or unclear.

### Procedures

First, the questionnaire was designed based on the literature review at home and abroad and consultation of experts.

Second, a pre-survey was conducted in February 2021 on the “i Guardian” Platform. Relying on the People’s Health Publishing House, the “i Guardian” platform is dedicated to building an authoritative information dissemination platform, including the service of conducting online questionnaires. Ten MSM completed the survey and the questionnaire was revised based on their feedback.

Third, from January to February 2021, researchers contacted MSM social organizations in six cities that had worked with us on multiple HIV-related projects and had good project implementation experience. Each MSM social organization in the six cities chose one or two people to conduct the survey, and then the researchers taught them how to conduct this study and helped them understand each question.

Fourth, the study launched from March 1 to April 30, 2021. The person in charge of the investigation sent the QR code of the questionnaire to the participants who came to the MSM social organization for consultation. After participants filled out the questionnaire anonymously on the “i Guardian” platform, they could share the QR code with qualified male companions around them via webpage, WeChat, QQ and other forms. Sample size was estimated by cross-sectional survey formula (N = Z^2^_1-a/2_P(1-P)/d^2^). Based on the PrEP utilization rate of 13.2% [17], 2526 people were needed. Considering the invalid response and other factors, the sample size was designed to be 2600.

Finally, the researchers checked the questionnaire database to ensure the completeness of the information (the authors were able to identify individual participants in the questionnaire database).

### Study variables

The questionnaire included demographic characteristics (age, ethnicity, domicile, education, etc.); basic knowledge of PrEP, willingness and use of PrEP; and sexual behavior (number of sexual partners, condom use, history of STDs, frequency of HIV testing, etc.). The primary outcome of this study was the rate of PrEP use among high-risk MSM. The secondary outcome of this study was the factors influencing the use of PrEP in high-risk MSM. Relevant definitions involving the outcome were shown below.

PrEP use was measured with a question, “Have you used PrEP?”. Answering “Yes” indicated that the participant had used PrEP.

The willingness to use PrEP was measured with a question, “Do you want to use PrEP if you are about to have a high-risk HIV behavior?”. Answering “Yes” indicated that the participant had the willingness to use PrEP.

PrEP knowledge awareness was defined by four questions [18], including (1) “What do you think is the function of HIV pre-exposure prophylaxis?”; (2) “Who do you think needs HIV pre-exposure prophylaxis?”; (3) “Do you know how to take pre-exposure prophylaxis?”; (4) “Do you think it is necessary to use condoms when having sex while taking pre-exposure prophylaxis?” The correct answers to the four questions were “HIV prevention”, “at risk for HIV infection”, “take daily” and “take two days before and after high-risk sex”, and “Yes”. Correct responses to all four questions would be recorded as PrEP knowledge awareness.

The definition of high-risk MSM included seven questions [19–22]. (1) Number of male sexual partners in the past six months was 10 or more; (2) Never used condoms when having sex with a male in the past six months; (3) Had commercial sex with MSM in the past six months. (4) Had male-to-male group sex frequently in the past 6 months (sex with 2 or more men at the same time); (5) Male sexual partner was HIV positive and had not received ART in the past 6 months; (6) Used drugs in the past 6 months (rush, methamphetamine, cocaine, ketamine, MDMA, etc.); (7) Having been diagnosed with an STD in the past 6 months. Meeting any of these conditions was considered as high risk MSM.

### Statistical analysis

This study focused on the use of PrEP by high-risk MSM who had heard of PrEP. MSM who had not heard of PrEP were not required to answer PrEP-related questions, so only those who had heard of PrEP were selected for subsequent statistical analysis. First, descriptive analysis (counts, percentages) was performed to describe demographic characteristics, awareness and use of PrEP, and sexual behavior among high-risk MSM. Then, factors influencing the use of PrEP among high-risk MSM who had heard of PrEP were analyzed by univariate and multivariate logistic regression. All statistical tests were analyzed with R 4.1.0, and *P*-value < 0.05 was considered statistically significant.

## Results

A total of 6147 questionnaires were collected. According to the inclusion criteria, invalid questionnaires were deleted, leaving 6035 valid questionnaires. The number of qualified questionnaires in the six cities of Beijing, Shenzhen, Chengdu, Changsha, Jinan and Nanjing were 2256, 809, 813, 408, 863 and 886, respectively. 4443 questionnaires were from WeChat, and the rest were filled in through other ways.

### High-risk MSM

Among 3882 (64.3%, 3882/6035) MSM had sex with men in the past six months, 6.2% (243) had more than 10 male sexual partners, 7.4% (287) never used condoms when having sex with a male, 7.4% (288) had commercial sex with MSM, 1.3% (48) had male-to-male group sex frequently, 1% (39) of the most recent male sexual partner was HIV positive and had not received ART, 45.3% (1760) took drugs, and 7.6% (292) had been diagnosed with STD (Table 1). According to the definition of high-risk MSM, there were 2188 high-risk MSM in this study. Then, only 1865 high-risk MSM had heard of PrEP.

**Table 1:** The characteristic of high-risk sexual behavior among MSM

Variables	Counts (N = 3882)	Percentage (%)
The numbers of men have had sex with^a^		
< 10	3639	93.8
≥ 10	243	6.2
Using of condoms during having sex with men^a^		
Never use	287	7.4
Sometimes use	1285	33.1
Use every time	2310	59.5
Had commercial sex with men^a^		
No	3594	92.6
Yes	288	7.4
Had group sex with men^a^		
Never	3285	84.6
Occasionally	549	14.1
Frequently	48	1.3
The HIV status of the male sexual partner^a^		
HIV negative	1760	45.3
Unclear	1633	42.1
HIV positive sexual partner and receiving antiviral therapy	450	11.6
HIV positive sexual partner and not receiving antiviral therapy	39	1.0
Used drugs^a^		
Yes	1760	45.3
No	2122	54.7
Had STDs^a^		
Yes	292	7.6
No	3587	92.4

^a^Refers to the time limit for these variables is within the last six months

### Characteristics of high-risk MSM

Among high-risk MSM who had heard of PrEP, median age was 32.2 years. 93.6% (1745/1865) of high-risk MSM who had heard of PrEP were Han nationality, 54.8% (1022) were domiciled in the city or province other than the one they lived in, 76.2% (1421) had a diploma degree or above, 75.8% (1413) were unmarried, 77.2% (1439/1865) had stable jobs, 62.2% (1160) had an average monthly income above 5000 RMB, 328(17.6) were bisexual, and 9.6% (180) had never been tested for HIV in the past year (Table 2).

**Table 2:** Baseline characteristics of MSM, high-risk MSM, and non-high-risk MSM

Variables	MSM n(%)	MSM had heard of PrEP				
		Total n(%)	High-risk MSM n(%)	Non-high-risk MSM n(%)	χ^2^	*P*-value^a^
Total (n)	6035	4806	1865	4170		
Age					10.709	0.013
18 ~	1432(23.7)	1126(23.4)	429(23.0)	697(23.7)		
26 ~	1358(22.5)	1134(23.6)	483(25.9)	651(22.1)		
31 ~	1327(22.0)	1081(22.5)	420(22.5)	661(22.5)		
> 36	1918(31.8)	1465(30.5)	533(28.6)	932(31.7)		
Ethnicity					6.479	0.011
Han nationality	5724(94.8)	4548(94.6)	1745(93.6)	2803(95.3)		
Other nationality	311(5.2)	258(5.4)	120(6.4)	138(4.7)		
Domicile					12.263	< 0.001
This city	2904(48.1)	2326(48.4)	843(45.2)	1483(50.4)		
Out of province/city	3131(51.9)	2480(51.6)	1022(54.8)	1458(49.6)		
Education					17.304	< 0.001
Junior high and below	627(10.4)	417(8.7)	128(6.9)	289(9.8)		
High School or Technical School	1184(19.6)	858(12.8)	316(16.9)	542(18.4)		
Diploma or Degree	3639(60.3)	3013(62.7)	1201(64.4)	1812(61.6)		
Master degree or above	585(9.7)	518(10.8)	220(11.8)	298(10.2)		
Heterosexual marital status					1.370	0.504
Unmarried	4406(73.0)	3637(75.7)	1413(75.8)	2224(75.6)		
Married	1017(16.9)	715(14.9)	267(14.3)	448(15.2)		
Divorced/widowed	612(10.1)	454(9.4)	185(9.9)	269(9.2)		
Job					11.092	0.011
Stable work	4466(74.0)	3611(75.1)	1439(77.2)	2172(73.8)		
Students	756(12.5)	603(12.6)	221(11.8)	382(13.0)		
Unstable work	696(11.5)	511(10.6)	185(9.9)	326(11.1)		
Retired	117(2.0)	81(1.7)	20(1.1)	61(2.1)		
Average monthly income					59.867	< 0.001
< 5000 RMB	2849(47.2)	2153(44.8)	705(37.8)	1448(49.2)		
≥ 5000 RMB	3186(52.8)	2653(55.2)	1160(62.2)	1493(50.8)		
Bisexuality					6.881	0.009
Yes	1285(21.3)	937(19.5)	328(17.6)	609(20.7)		
No	4749(78.7)	3869(80.5)	1537(82.4)	2332(79.3)		
Number of HIV tests in the last year					108.360	< 0.001
0	763(12.6)	557(11.6)	180(9.6)	377(12.8)		
1	1391(23.1)	1073(22.3)	373(20.0)	700(23.8)		
2	1283(21.3)	1072(22.3)	390(20.9)	682(23.3)		
3	876(14.5)	751(15.6)	292(15.7)	459(15.6)		
4	848(14.1)	650(13.6)	238(12.8)	412(14.0)		
≥ 5	871(14.4)	700(14.6)	392(21.0)	308(10.5)		

^a^*P*-values for comparison of high-risk MSM had heard of PrEP and non-high-risk MSM had heard of PrEP

### Knowledge awareness, willingness, uptake of PrEP among high-risk MSM

Among 1865 high-risk MSM who had heard of PrEP, 95.9% (1788) thought PrEP was to prevent AIDS, 90.4% (1686) thought people who at high risk of HIV infection needed PrEP, 51.3% (956) knew that PrEP could be taken daily or 74.2% (1383) knew that PrEP could be taken two days before and after high-risk sexual behaviors, and 90.1% (1681) knew they needed to use condoms while taking PrEP. According to the definition of PrEP knowledge awareness, 24.7% (460) knew the basic knowledge of PrEP. 38.4% (717) participants knew PrEP from MSM social organizations, and 36.2% (676) from the Internet. 96.7% (1804) of MSM were willing to use PrEP if they had high-risk sexual behavior. 43.3% (808) were reluctant to use PrEP because of the price of the drug, 18.8% (351) were reluctant to use PrEP because they were worried about the effects, and 19.6% (365) were reluctant to use PrEP because they were worried about the side effects. 46.6% (869) of high-risk MSM consulted PrEP and 22.4% (417) had used PrEP (Table 3).

**Table 3:** Knowledge awareness and uptake of PrEP among high-risk MSM and non-high-risk MSM

Variables	MSM n(%)	High-risk MSM n(%)	Non-high-risk MSM n(%)	χ^2^	*P*-value^a^
Total (n)	4806	1865	2941		
Role of PrEP^b^				40.136	< 0.001
HIV treatment	232(4.8)	46(2.5)	186(6.3)		
HIV prevention	4480(93.2)	1788(95.9)	2692(91.5)		
Prevent STDs	62(1.3)	23(1.2)	39(1.3)		
Do not know	32(0.7)	8(0.4)	24(0.9)		
PrEP users^b^				25.863	< 0.001
Persons living with HIV	245(5.1)	61(3.3)	184(6.3)		
People at high risk of HIV infection	4236(88.1)	1686(90.4)	2550(86.7)		
General public	270(5.6)	104(5.6)	166(5.6)		
Do not know	55(1.2)	14(0.7)	41(1.4)		
PrEP dosing regimen (multiple-choice)^b^					
Take it daily	2392(49.8)	956(51.3)	1436(48.8)	2.606	0.106
Take two days before and after high-risk sexual behavior	3448(71.7)	1383(74.2)	2056(70.2)	8.552	0.003
Do not know	193(4.0)	68(3.6)	125(4.2)	0.930	0.335
Condom use while taking PrEP^b^				55.535	< 0.001
Yes	4492(93.5)	1681(90.1)	2811(95.6)		
No	199(4.1)	118(6.3)	81(2.7)		
Do not know	115(2.4)	66(3.6)	49(1.7)		
Sources to get information of PrEP^b^				23.853	< 0.001
MSM community organizations	1717(35.7)	717(38.4)	1000(34.0)		
Internet	1715(35.7)	676(36.2)	1039(35.3)		
CDC	839(17.5)	267(14.4)	572(19.5)		
Others	535(11.1)	205(11.0)	330(11.2)		
Willingness to take PrEP^b^				2.336	0.126
Yes	4672(97.2)	1804(96.7)	2868(97.5)		
No	134(2.8)	61(3.3)	73(2.5)		
Main barriers to willingness to use PrEP^b^				38.057	< 0.001
Effectiveness	1045(21.7)	351(18.8)	694(23.6)		
Drug price	1834(38.2)	808(43.3)	1026(34.9)		
Side effects	1021(21.2)	365(19.6)	656(22.3)		
Frequency and duration of medication	628(13.1)	233(12.5)	395(13.4)		
Others	278(5.8)	108(5.8)	170(5.8)		
Consulted PrEP^b^				3.086	0.079
Yes	2162(45.0)	869(46.6)	1293(44.0		
No	2644(55.0)	996(53.4)	1648(56.0)		
Used PrEP^b^					
Yes	848(17.6)	417(22.4)			
No	3958(82.4)	1448(77.6)			

^a^P-values for comparison of high-risk MSM had heard of PrEP and non-high-risk MSM had heard of PrEP

^b^The prerequisite for this variable was to have heard of PrEP

### Related factors of using PrEP among high-risk MSM

Univariate logistic regression analysis showed that age, job, number of HIV tests in the past year, PrEP knowledge awareness, ways of knowing PrEP, willingness to use PrEP, barriers to willingness to use PrEP, and PrEP counseling were associated with PrEP use (*P* < 0.05). The results of multivariate logistic regression model analysis showed that the related factors of the PrEP use included age [26 years and above (*OR* = 1.86, 95% confidence interval (CI 1.17 ~ 2.99), 31 years and above (*OR* = 1.98, 95%*CI* 1.23 ~ 3.24), and over 36 years old (*OR* = 2.18, 95%*CI* 1.38 ~ 3.50)],master degree or above (*OR* = 2.37, 95%CI 1.2 ~ 4.72), erratic work (*OR* = 1.86, 95%CI 1.16 ~ 2.96), HIV testing 5 or more times in the past year (*OR* = 3.09, 95%*CI* 1.65 ~ 6.04), PrEP knowledge awareness (*OR* = 1.90, 95%*CI* 1.41 ~ 2.55), sources to get information of PrEP [CDC (*OR* = 1.75, 95%*CI* 1.17 ~ 2.60), others (*OR* = 1.75, 95%*CI* 1.13 ~ 2.73)], other barriers to PrEP willingness (*OR* = 0.23, 95%*CI* 0.08 ~ 0.56) and consulted PrEP (*OR* = 22.05, 95%*CI* 14.87 ~ 33.91) (Table 4).

**Table 4:** Logistic regression analysis to the uptake of PrEP among high-risk MSM

Variables	High-risk MSM	Used PrEP, n(%)	Univariate analysis^a^		Multivariate analysis^b^	
			*OR* (95%CI)	*P*-value^c^	OR (95%CI)	*P*-value^c^
Total (n)	1865	417(22.4)				
Age						
18 ~	429	64(14.9)	1.00		1.00	
26 ~	483	111(23.0)	1.70 (1.22 ~ 2.40)	0.002	1.86(1.17 ~ 2.99)	0.009
31 ~	420	103(24.5)	1.85 (1.31 ~ 2.63)	< 0.001	1.98(1.23 ~ 3.24)	0.006
> 36	533	139(26.1)	2.01 (1.45 ~ 2.81)	< 0.001	2.18(1.38 ~ 3.50)	0.001
Education						
Junior high and below	128	29(22.6)	1.00		1.00	
High school or technical school	316	54(17.1)	0.70 (0.43 ~ 1.18)	0.174	0.95(0.52 ~ 1.76)	0.871
Diploma or degree	1201	263(21.9)	0.96(0.63 ~ 1.50)	0.844	1.64(0.94 ~ 2.92)	0.084
Master degree or above	220	71(32.3)	1.63(0.99 ~ 2.71)	0.057	2.37(1.21 ~ 4.72)	0.013
Job						
Stable work	1439	321(22.3)	1.00		1.00	
Students	221	40(18.1)	0.77(0.53 ~ 1.10)	0.159	1.12(0.63 ~ 1.99)	0.705
Unstable work	185	54(29.2)	1.44(1.02 ~ 2.01)	0.037	1.86(1.16 ~ 2.96)	0.009
Retired	20	2(10.0)	0.39(0.06 ~ 1.35)	0.204	0.31(0.05 ~ 1.30)	0.156
Average monthly income						
< 5000 RMB	705	144(20.4)	1.00		1.00	
≥ 5000 RMB	1160	273(23.5)	1.20(0.96 ~ 1.51)	0.118	0.88(0.62 ~ 1.24)	0.457
Number of HIV tests in the last year						
0	180	16(8.9)	1.00		1.00	
1	373	43(11.5)	1.34(0.74 ~ 2.51)	0.347	0.86(0.43 ~ 1.75)	0.667
2	390	58(14.9)	1.79(1.02 ~ 3.31)	0.051	1.12(0.58 ~ 2.25)	0.740
3	292	67(22.9)	3.05(1.75 ~ 5.63)	< 0.001	1.66(0.85 ~ 3.34)	0.145
4	238	67(28.2)	4.02(2.29 ~ 7.44)	< 0.001	1.76(0.90 ~ 3.59)	0.108
≥ 5	392	166(42.3)	7.53(4.46 ~ 13.53)	< 0.001	3.09(1.65 ~ 6.04)	< 0.001
PrEP knowledge awareness						
Yes	460	168(36.5)	2.67(2.11 ~ 3.38)	< 0.001	1.90(1.41 ~ 2.55)	< 0.001
No	1405	249(17.7)	1.00		1.00	
Sources to get information of PrEP						
MSM community organizations	717	143(19.9)	1.00		1.00	
Internet	676	122(18.0)	0.88(0.67 ~ 1.15)	0.367	1.17(0.84 ~ 1.63)	0.363
CDC	267	86(32.2)	1.91(1.39 ~ 2.61)	< 0.001	1.75(1.17 ~ 2.60)	0.006
Others	205	66(32.2)	1.91(1.35 ~ 2.69)	< 0.001	1.75(1.13 ~ 2.73)	0.013
Willingness to use PrEP						
Yes	1804	412(22.8)	3.31(1.45 ~ 9.55)	0.011	0.88(0.32 ~ 2.86)	0.810
No	61	5(8.2)	1.00		1.00	
The main barriers to PrEP willingness						
Effectiveness	351	97(27.6)	1.38(1.03 ~ 1.84)	0.028	1.43(0.99 ~ 2.05)	0.056
Drug price	808	175(21.6)	1.00		1.00	
Side effects	365	83(22.7)	1.06(0.79 ~ 1.43)	0.679	1.02(0.70 ~ 1.48)	0.907
Frequency and duration of medication	233	56(24.0)	1.14(0.81 ~ 1.61)	0.442	1.17(0.75 ~ 1.81)	0.482
Others	108	6(5.6)	0.21(0.08 ~ 0.45)	< 0.001	0.23(0.08 ~ 0.56)	0.003
Consulted PrEP						
Yes	869	388(44.6)	26.90(18.48 ~ 40.66)	< 0.001	22.05(14.87 ~ 33.91)	< 0.001
No	996	29(2.9)	1.00		1.00	

*OR* odds ratio; *CI* confidence Interval

^**a**^Univariable logistic regression model

^**b**^Multivariable logistic regression model

^**c**^Wald test

## Discussion

Although PrEP is an important measure to HIV prevention in high-risk MSM, the rate of PrEP use was low (22.4%). Because there were no studies in the literature on the use of PrEP in high-risk MSM, PrEP use was relatively high in high-risk MSM compared to MSM. For example, the rate (22.4%) was higher than a cross-sectional survey of PrEP use among MSM in 34 cities in China (1.2% had used PrEP) [23] and a cohort study implemented in Harbin, China (0.7% of MSM used PrEP at baseline survey) [24]. This was because high-risk MSM were more sexually active and had a greater need for PrEP and therefore used more of it. In addition, high-risk MSM had higher knowledge of the role, suitable crowd, dosing regimen of PrEP than non-high-risk MSM, and PrEP knowledge was a factor promoting PrEP use among high-risk MSM, considering that PrEP use also be higher among high-risk MSM due to better knowledge of PrEP. But, the rate of PrEP use among High-risk MSM (22.4%) was lower than a cross-sectional analysis of MSM who had problematic sexual behavior within the United Kingdom (34%) [25] and a descriptive analysis of MSM in the National HIV Behavior Surveillance (NHBS) system in Boston (50.2%) [26]. Compared with the rate of PrEP use in developed countries, the promotion of PrEP in China was still insufficient. Further efforts are still needed to promote PrEP so that more MSM who need it can use it in a timely manner. Furthermore, the results suggested that high-risk MSM presented a high willingness to use, low knowledge awareness and use of PrEP (96.7% were willing to use PrEP, 24.7% had PrEP knowledge awareness and 22.4% used PrEP). It became a serious challenge to increase the knowledge and use of PrEP among high-risk MSM to meet their high willingness.

Factors influencing PrEP use among high-risk MSM included age, education, job, number of HIV tests in the past year, knowledge awareness of PrEP, and whether or not consulted PrEP. The results showed that high-risk MSM with unstable jobs and higher education were more likely to use PrEP, which was consistent with the results of Siyan Yi et al. [27]. Since MSM with higher education might have easier access to health-related information [28]. Besides, the results of Guan Y [23] showed that subjective barriers to using PrEP included unemployment and objective barriers included high costs. In response to the unemployment issue, this study complemented that high-risk MSM without regular jobs used more PrEP than MSM with regular jobs, considering that MSM with unstable jobs had more frequent high-risk sexual behaviors. In addition, in order to deal with the issue of high costs being an objective barrier to the use of PrEP, this study had made a different finding. This study found that monthly income was not an influence on PrEP use in high-risk MSM, which was inconsistent with the study by Brooks, R. A. et al. [29]. The current price of PrEP drugs in China fluctuated greatly. Users only needed to take 4 pills in a course of PrEP for an average of 25–43 yuan (RMB). The low price made more MSM willing to use it. The THRIVE program provided PrEP to higher proportions of PrEP-eligible persons than current national estimates, however PrEP use disparities persist, suggesting a need to increase PrEP coverage [30]. It is suggested that improving the coverage of PrEP is one of the key issues to solve the differences in the use of PrEP. However, online services for selling PrEP drugs have flourished in China, with multiple online purchasing platforms available to purchase PrEP, further helping to increase PrEP accessibility and expand coverage.

A systematic review of 23 studies showed that younger MSM were more likely to take PrEP [31], in contrast to the results of Yu Liu et al. [32] and our findings. Older high-risk MSM had more exposure to PrEP and might have better knowledge about PrEP. Combined with the results of the multivariate logistic regression analysis, knowledge awareness promoted PrEP use in high-risk MSM, so that older MSM had more use of PrEP. In addition, participants who had consulted PrEP were more likely to use PrEP. MSM who have consulted PrEP would know more information about PrEP, on the other hand, it might also be due to the fact that MSM who had consulted PrEP had a higher willingness to use PrEP. The accompanying measures and one-on-one counseling by a trained counselor could increase the effectiveness of this PrEP program [33]. Therefore, valid and accurate information from PrEP counselors is needed.

The results showed that as the number of HIV tests increased, more high-risk MSM used PrEP, consistent with the findings of the other two studies [34, 35]. Because patients seeking PrEP were screened for HIV before starting therapy as well as every 3 months after beginning treatment with PrEP [36]. In addition, high-risk MSM had more frequent high-risk sexual behavior, and going for frequent HIV testing reflected both the importance they placed on their health and the fear of HIV infection. Early testing can reduce complications of HIV infection and reduce the risk of transmission. It is estimated that 40% of new infections of HIV are transmitted by those who are not aware of their HIV diagnosis [37]. The day of HIV testing was the critical moment for people with high HIV exposure risk to start PrEP [38]. It was suggested that advertising PrEP during HIV testing or HIV self-testing was a very good way to increase the use of PrEP. MSM who do not test frequently for HIV are less likely to be aware of PrEP, test for sexually transmitted infections, or use condoms [32]. 6.3% of high-risk MSM thought that condom use was not required for sex while taking PrEP, consistent with the findings of Braksmajer. A. and Ahouada. C. [18, 39]. Although the percentage was relatively small, this issue should not be ignored. After all, PrEP is not absolutely 100% effective in HIV prevention. Furthermore, in Brazil, MSM reported that learning about PrEP online positively influenced their willingness to use it [40]. And a web-based survey in Latin America, willingness to use PrEP was found to be high and directly related to PrEP awareness [41]. The results of these two studies suggested that MSM had higher acceptability when PrEP was promoted on the Web, so PrEP promotion can be enhanced on the Web, with particular emphasis on condom use when promoting PrEP.

In this study, MSM were recruited through an MSM social organization in an online “snowballing” way where participants anonymously completed questionnaires and shared it with their eligible male peers. This "snowballing" method has the disadvantage of not being able to track the entire process, such as not knowing how many times it has been rolled, how many people were contacted each time and how many people responded, and only knowing the total number of questionnaires at the end. This shortage does not affect the quality of the questionnaire. Besides, through this online questionnaire sharing, MSM who were more interested in the topic would choose to answer. In addition, MSM had recalling bias when completing the questionnaire, but the researchers limited the questions to a certain period of time, such as "in the past six months" to reduce bias.

## Conclusion

In conclusion, the rate of PrEP use in high-risk MSM was relatively low. And high-risk MSM showed high willingness to use PrEP, low knowledge awareness, and low usage. Therefore, government and health authorities should continue to strengthen MSM awareness of PrEP and condoms. How to reduce the gap between high willingness and low usage is the next step to be studied. PrEP was more used by high-risk MSM with unstable jobs, higher education, frequent HIV testing, and PrEP counseling. However, it is also worth considering whether these variables have a direct effect on PrEP use, or an indirect effect on PrEP use through knowledge of PrEP or other factors.

## Data Availability

The data generated for this study are not publicly available but are available from the corresponding author on reasonable request.
